# Electrochemical Sensor for Bilirubin Detection Using Screen Printed Electrodes Functionalized with Carbon Nanotubes and Graphene

**DOI:** 10.3390/s18030800

**Published:** 2018-03-07

**Authors:** Madasamy Thangamuthu, Willimann Eric Gabriel, Christian Santschi, Olivier J. F. Martin

**Affiliations:** Nanophotonics and Metrology Laboratory (NAM), Swiss Federal Institute of Technology Lausanne (EPFL), 1015 Lausanne, Switzerland; eric.willimann@epfl.ch (W.E.G.); christian.santschi@epfl.ch (Ch.S.); olivier.martin@epfl.ch (O.J.F.M.)

**Keywords:** screen printed electrode (SPE), bilirubin, electrochemical sensor, carbon nanotubes (CNT), graphene, nanomaterials, electrochemical analysis

## Abstract

Practice oriented point-of-care diagnostics require easy-to-handle, miniaturized, and low-cost analytical tools. In a novel approach, screen printed carbon electrodes (SPEs), which were functionalized with nanomaterials, are employed for selective measurements of bilirubin, which is an important biomarker for jaundice. Multi-walled carbon nanotubes (MWCNT) and graphene separately deposited on SPEs provide the core of an electrochemical sensor for bilirubin. The electrocatalytic activity towards bilirubin oxidation (bilirubin to biliverdin) was observed at +0.25 V. In addition, a further peak corresponding to the electrochemical conversion of biliverdin into purpurin appeared at +0.48 V. When compared to MWCNT, the graphene type shows a 3-fold lower detection limit (0.3 ± 0.022 nM and 0.1 ± 0.018 nM, respectively), moreover, the graphene type exhibits a larger linear range (0.1–600 µM) than MWCNT (0.5–500 µM) with a two-fold better sensitivity, i.e., 30 nA µM^−1^ cm^−2^, and 15 nA µM^−1^ cm^−2^, respectively. The viability is validated through measurements of bilirubin in blood serum samples and the selectivity is ensured by inhibiting common interfering biological substrates using an ionic nafion membrane. The presented approach enables the design and implementation of low cost and miniaturized electrochemical sensors.

## 1. Introduction

Bilirubin (BR, a yellow colored pigment) is a by-product stemming from the natural breakdown of old red blood cells, which is a catabolic process that is necessary to remove waste products [1]. BR is classified into two types related to the conjugation with glucuronic acid *viz.* direct (conjugated) and indirect (unconjugated). Conjugated BR forms a water soluble complex with glucuronic acid whereas the free unconjugated BR binds with albumin and becomes soluble in water [2]. A tiny fraction of free BR (not bound with albumin) is an important indicator for bilirubin toxicity [3]. The normal concentration of direct bilirubin in human blood is around 1–5 μM (0.06–0.3 mg/dL) and the total BR concentration is around 25 μM (<1.23 mg/dL) [4]. The total BR concentration in the blood increases when the liver is not excreting properly BR, which causes jaundice that is associated with liver diseases [5]. It can also cause brain damages and other serious problems in new-born babies. On the other hand, low level of BR indicates anaemia and coronary heart diseases [6]. Therefore, the accurate quantification of the total BR concentration in blood becomes clinically very important, and, hence, the development of analytical methods for BR detection is of great interest.

Several methods have been developed to detect BR, with direct spectroscopic measurements and diazo reaction being the most common methods [7,8]. Spectroscopic methods suffer from interference with other proteins and the accuracy in the diazo method is uncertain since the reaction rate is pH dependent. Other known and commercially available methods, such as polarography [9], fluorometry [10,11], and colorimetric assay kits [Sigma Aldrich-MAK126, Biovision-K553-100 and Cell Biolabs-MET5010] are expensive and less selective. Recently, electrochemical sensors have been proven to be suitable alternatives since they are simple and selective. In particular, bilirubin oxidase (BOx) immobilized electrodes have been studied for the selective determination of BR [12,13,14,15,16]. In the presence of molecular oxygen, BOx oxidizes the BR into biliverdin and the concentration of BR is determined indirectly by measuring the depletion of molecular oxygen [17] or the generation of hydrogen peroxide [18]. In this condition, other electroactive species are also possibly involved in the reaction and the hydrogen peroxide generation is stoichiometrically not equal to BR. In addition, BOx is expensive and not stable under ambient conditions. To this end, researchers focus on nanomaterials to develop stable non-enzymatic and cost effective electrochemical BR sensors.

Electrodes that are modified with nanomaterials, such as metal nanoparticles [19], nanostructural conducting polymers (molecularly imprinted polymer) [20,21,22,23], carbon nanotubes [24,25], graphene, and their composites [26,27] have been reported as BR sensors. Those nanomaterials are well known for their efficiency to improve the electrocatalytic performance of the sensors by increasing the effective surface area. Even if the non-enzymatic BR sensors are stable enough, they might not be adapted for point-of-care analysis since small size and low cost electrodes are required to measure the BR concentration in a sample droplet. Recently, screen printed carbon electrodes (SPEs) have been proven to be a suitable alternative for expensive conventional electrodes. They fulfil not only low-cost and small size requirements, but they also provide practical advantages, such as low detection limit, high flexibility, fast response time, high reproducibility [28,29,30], and have also great potential for miniaturization and mass production. 

Hence, in the present study, we have chosen SPEs to develop a point-of-care assay for BR. Multi-walled carbon nanotubes (MWCNT) and graphene are especially attractive for sensor fabrication as they have fascinating properties, such as large surface area, good mechanical and electrical properties, and excellent chemical/thermal stability [31,32]. Thus, we have deposited MWCNT or graphene separately on SPEs and studied the resulting enhancement of the electroanalytical parameters, such as lower detection limit, wider linear range, and higher sensitivity. Electrochemical oxidation experiments that were carried out with graphene or MWCNT coated SPEs revealed a better performance for graphene. The viability of the sensors was validated by measuring the BR concentration in blood serum samples and the selectivity was ensured by inhibiting common interfering biological substrates using an ionic nafion membrane.

## 2. Materials and Methods

### 2.1. Chemicals and Reagents

All of the chemicals and reagents were purchased from Sigma-Aldrich (Buchs, Switzerland) at their highest available purity and used as received without further purification; Bilirubin (BR), dimethyl sulfoxide (DMSO), potassium ferricyanide (K_3_[Fe(CN)_6_]), human serum (male AB plasma USA origin, Buchs, Switzerland), nafion 0.5% wt, potassium chloride (KCl), sulfuric acid (H_2_SO_4_), COOH-functionalized multi-walled carbon nanotubes (MWCNT, 19 nm diameter), and graphene oxide (GO) dispersed in water (1 mg mL^−1^). The stock solution of BR was diluted in 10 mM DMSO solvent. Phosphate Buffered Saline (0.1 M PBS, pH 7.2) from Gibco (ThermoFisher scientific, Reinach, Switzerland) was used for dilution and the experiments were performed under dark conditions, as BR is photosensitive. Milli-Q water was used for preparing PBS solution. 

### 2.2. Measurement of BR in Blood Serum Samples 

A commercially available lyophilized human blood serum sample was reconstituted in 0.1 M PBS (pH 7.2) and diluted 10 times separately. Determination of the BR concentration was performed using the amperometric technique by placing 30 μL of the sample over MWCNT or graphene functionalized SPE surface. The unknown concentration of BR was quantified from the standard addition calibration curve and was multiplied by the dilution factor.

### 2.3. Instrumentation and Measurements 

Cyclic voltammetric and amperometric experiments were performed using the CHI 1240B electrochemical workstation (CH Instruments, Austin, TX, USA) with a conventional three electrode system. A three electrode type of SPE (TE100, CH Instruments) consisting of a Ag/AgCl reference electrode, a carbon counter electrode, and a carbon working electrode modified with MWCNT or electrochemically reduced graphene oxide (Er-GR) was used as sensing element. The surface area of the working electrode was 0.071 cm^2^. The electrode was equilibrated in 0.1 M PBS electrolyte by performing cyclic voltammetry until the voltammogram became constant. Cyclic voltammetric characterizations were performed in the voltage range between −1 to +1 at a scan rate of 50 mV s^−1^. Amperometric (time vs. current) measurements were done for 300 s and the stable current at 200 s was used to study the performance of the sensor. The morphological scanning electron microscope (SEM) images were obtained using a Zeiss Merlin field emission SEM (Carl Zeiss, Jena, Germany) at 2 keV. 

### 2.4. Sensor Fabrication 

**Figure 1:**
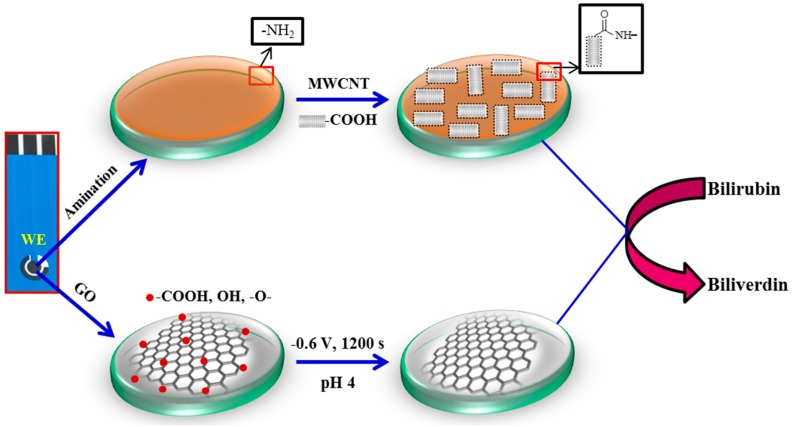
Schematic drawing of the preparation of the multi-walled carbon nanotubes (MWCNT) based (top row) or electrochemically reduced graphene oxide (Er-GR) based (bottom row) bilirubin sensors.

Before nanomaterials deposition, SPE was pre-treated, as described in our earlier report [33], to remove the organic ink constituents or contaminants and to increase the surface functionalities. Then, the amine (-NH_2_) functionalized carbon surface was formed by electro-oxidation in 0.1 M ammonium carbamate solution applying a constant potential of 1.0 V for 10 min. MWCNT coated SPEs were obtained by drop casting 6 μL of MWCNT solution (1 mg mL^−1^) with subsequent drying in air. As a result, MWCNT covalently bond with the amide bonds on the carbon surface (Figure 1). The GO solution was drop casted onto the bare carbon surface (no NH_2_ functionalization), dried in air, and subsequently electrochemically reduced in 0.1 M PBS and KCl (pH 4.0) applying a potential of −0.6 V for 1200 s. During this process, -COOH, -OH, and oxygen functional groups are reduced, providing electrochemically reduced graphene oxide modified SPE, denoted as Er-GR-SPE.

## 3. Results

### 3.1. Morphological Characterization 

**Figure 2:**
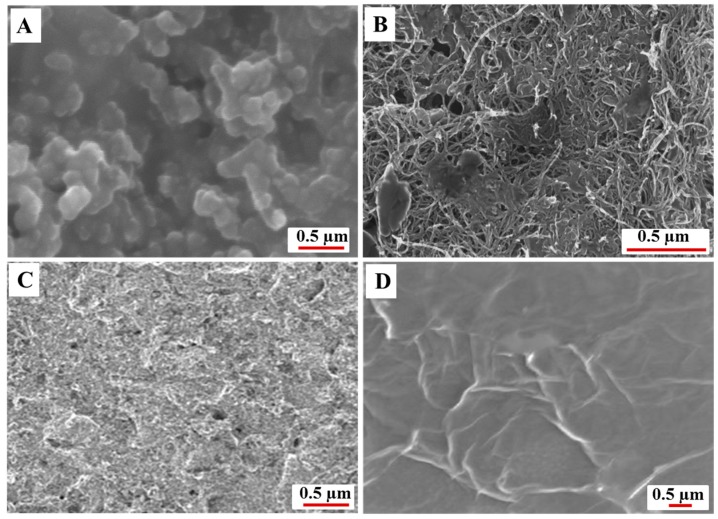
SEM images of the morphology of the (**A**) screen printed carbon electrodes (SPE), (**B**) MWCNT-SPE, and graphene oxide (GO)-SPE before (**C**) and after electrochemical reduction (**D**).

**Figure 3:**
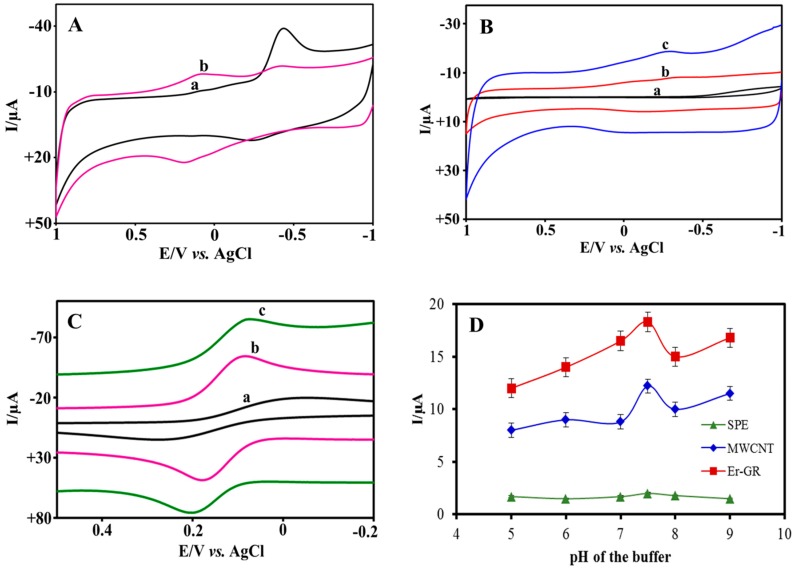
(**A**) Electrochemical responses of the GO-SPE (a) before and (b) after electrochemical reduction in 0.1 M PBS (pH 7.2) at a scan rate of 50 mV s^−1^. (**B**) Typical electrochemical responses of the (a) bare SPE, (b) MWCNT–SPE, and (c) Er-GR-SPE in 0.1 M PBS (pH 7.2) and (**C**) in 0.1 M PBS containing 2.5 mM K_3_[Fe(CN)_6_] at a scan rate of 50 mV s^−1^. (**D**) Effect of the pH value on the voltammetric current responses (at +0.48 V) of the bare SPE, MWCNT-SPE, and Er-GR-SPE in 0.1 M PBS containing 100 µM BR at scan rate of 50 mV s^−1^.

The SEM images showing the morphology of bare SPE, MWCNT–SPE, and Er-GR-SPE are displayed in Figure 2. It can be seen that the surface of the bare SPE is porous (Figure 2A). Figure 2B shows the successful functionalization of MWCNT on -NH_2_ functionalized SPE. Figure 2C,D show the GO modified SPE before and after electrochemical reduction. A clear change in the morphology observed after electrochemical reduction suggests a successful reduction of -COOH, -OH, and oxygen functional groups. It is also evident from the cyclic voltammograms that are applied on the GO-SPE before and after electrochemical reduction (Figure 3A) that the reduction peak observed at −0.5 V (Vs. Ag/AgCl) corresponds to the electrochemical reduction of GO [25]. Furthermore, a stable redox peaks around +0.2 V was observed after 500 cycles. The reduction of GO is monitored by tracking the peak at −0.5 V, which disappears upon complete reduction. 

### 3.2. Electrochemical Characterization 

The electrochemical behavior of the MWCNT-SPE and Er-GR-SPE were investigated using cyclic voltammetry in 0.1 M PBS at a scan rate of 50 mV s^−1^, as shown in Figure 3B. The observed voltammograms show a higher electrochemical current for Er-GR modified electrodes when compared to MWCNT modified electrodes, which is attributed to the higher electrical conductivity of the Er-GR-SPE, resulting from the intrinsic electron mobility of the Er-GR [34]. Furthermore, the electron transfer properties of these electrodes were investigated in the presence of a Fe^3+^/Fe^2+^ redox probe, as shown in Figure 3C exhibiting the same trend. 

### 3.3. Effect of pH 

**Figure A1:**
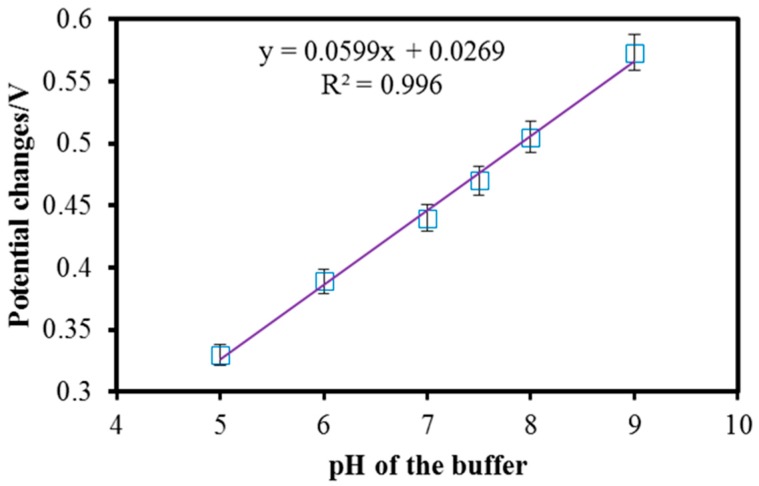
Effect of the pH value on the voltammetric potential responses of Er-GR-SPE in 0.1 M PBS containing 100 µM BR at scan rate of 50 mV s^−1^.

The influence of the pH value on the oxidation potential and electrochemical current responses of the bare SPE, Er-GR-SPE, and MWCNT-SPE was investigated in buffered solutions containing 100 µM BR. The pH values were altered from 5 to 9 in steps of 1.0. The oxidation potential of BR changes, while increasing the pH, as shown in Figure A1 (Appendix), and the obtained slope value (ca. 0.059) indicates that electrochemical oxidation of BR follows Nerst equation. Furthermore, the voltammetric current responses vs. pH were recorded for +0.48 V as shown in Figure 3D. The maximum current response on each electrode was observed at pH 7.5. Hence, the subsequent experiments were carried out under optimized conditions maintaining the pH at ~7.5. Nanomaterial modified SPEs show higher current responses when compared to bare SPE, which can be attributed to the larger surface area offered by the nanomaterials and allows for more BR to bind on the surface, thus enhancing the electron transport rate.

### 3.4. Electrocatalytic Oxidation of BR 

**Figure 4:**
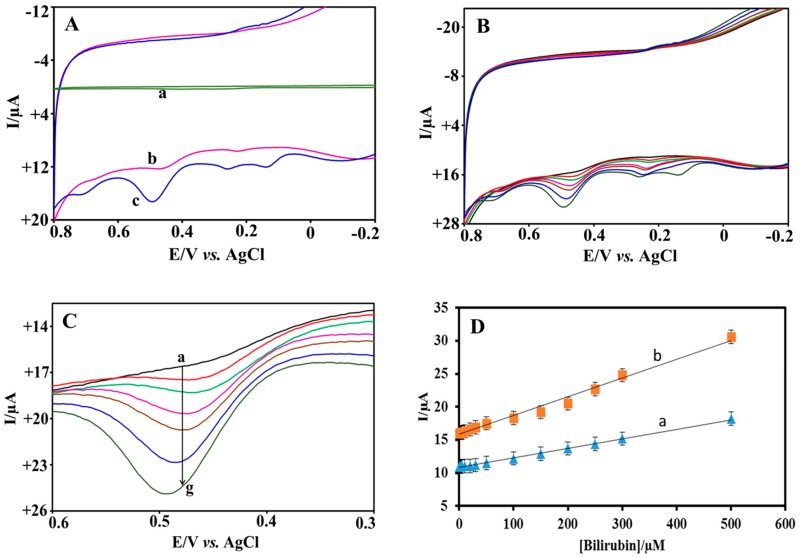
(**A**) Electrochemical responses of the (a) bare SPE, (b) MWCNT-SPE, and (c) Er-GR-SPE in the presence of 100 µM BR in 0.1 M PBS (pH 7.2) at a scan rate of 50 mV s^−1^. (**B**) Electrochemical responses of the Er-GR-SPE in the presence of (a) 10, (b) 50, (c) 100, (d) 150, (e) 200, (f) 250, and (g) 300 µM of BR in 0.1 M PBS (pH 7.2) at a scan rate of 50 mV s^−1^. (**C**) Zoomed in to +0.48 V. (**D**) Calibration plot for BR using MWCNT-SPE (a) Er-GR-SPE (b).

The electrochemical oxidation of BR on bare SPE, Er-GR-SPE, and MWCNT-SPE was investigated in the presence of 100 µM BR in 0.1 M PBS (pH 7.2) at a scan rate of 50 mV s^−1^, as shown in Figure 4A. Two oxidation peaks were observed, one at +0.25 V, corresponding to the oxidation of BR to biliverdin (reaction 1), and another at +0.48 V (reaction 2), corresponding to the oxidation of biliverdin to purpurine. Oxidation of purpurine to choletelin (reaction 3) at higher potential was also observed, e.g., on Er-GR-SPE. A small peak at +0.15 V is ignored as it is not significant because this peak does not appear at lower BR concentration, as is evident from Figure 4B. Control experiments, in the absence of BR, did not show any oxidation response for the different electrodes. Figure 4B shows the electrochemical response of the Er-GR-SPE, while increasing the concentration of BR in the range from 10 to 300 µM, which covers the BR levels in human plasma for many patho/physiological situations [3]. The most pronounced oxidation response is observed at +0.48 V, and therefore the concentration of BR is measured by monitoring the oxidation response at that potential (Figure 4C). The performances of the MWCNT-SPE and Er-GR-SPE for various concentrations of BR were studied using amperometric technique by applying constant potential of +0.48 V. A linear increase in oxidation current with increasing BR concentrations was observed and the anodic currents vs. BR concentrations were plotted, as shown in Figure 4D. The obtained calibration curve for MWCNT-SPE exhibits a dynamic linear range over the BR concentrations from 0.5–500 µM with a detection limit of 0.3 ± 0.022 nM and sensitivity of 15 nA µM^−1^ cm^−2^, whereas Er-GR-SPE shows a three-fold lower detection limit (0.1 ± 0.018 nM), larger linear range (0.1–600 µM) with a two-fold better sensitivity, i.e., 30 nA µM^−1^ cm^−2^.

### 3.5. Specificity 

**Figure 5:**
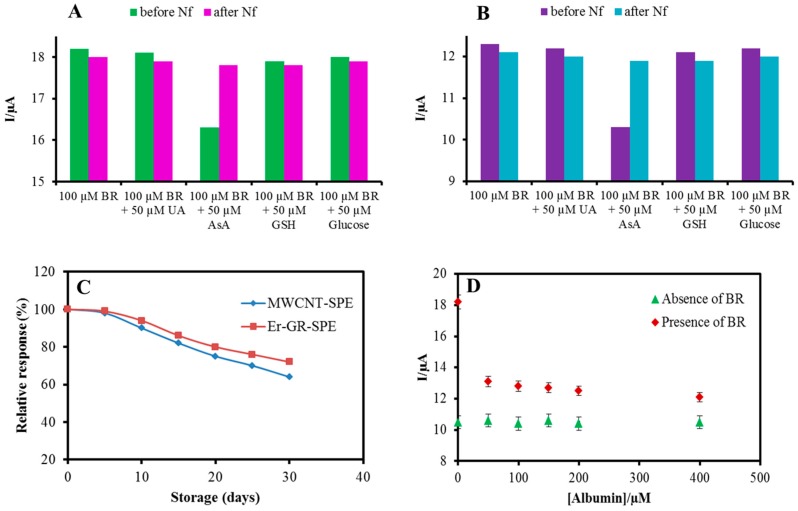
Histograms representing Bilirubin (BR) oxidation current for the Er-GR-SPE (**A**) and MWCNT-SPE (**B**) before and after nafion coating, upon addition of 50 µM of UA, AsA, GSH and glucose in 0.1 M PBS containing 100 µM BR. (**C**) Relative BR oxidation current responses of the MWCNT-SPE and Er-GR-SPE over long term storage. (**D**) Electrochemical current responses of the Er-GR-SPE at +0.48 V in 0.1 M PBS containing 0, 50, 100, 150, 200, and 400 µM of albumin in the absence and presence of 100 µM BR.

Since the oxidation potential of BR is relatively high positive, common interfering substrates, such as glucose, ascorbic acid (AsA), uric acid, and glutathione could interfere with the BR measurement. The selectivity of the MWCNT-SPEs and Er-GR-SPEs was investigated by monitoring the change in oxidation current using amperometric technique in 0.1 M PBS for 100 µM BR at +0.48 V upon the addition of 50 µM of each interfering substrates. The addition of uric acid, glutathione, and glucose did not significantly change the response of the sensors. However, addition of AsA steeply reduces the current response of the MWCNT and Er-GR-SPEs due to its strong reduction properties as shown in Figure 5A,B. To eliminate the interference effect of AsA, the electrodes were covered with a thin nafion membrane by placing a droplet of nafion resin solution (0.05% wt) onto the substrate with subsequent drying under ambient conditions. This procedure forms a thin, porous, and negatively charged membrane on top of the electrodes, which considerably reduces the interference with AsA, as shown in Figure 5A,B. As a positive side effect, nafion coating also preserves the electrodes from fouling that is caused by non-specific adsorption of proteins and other molecules that are typically present in biological samples. 

### 3.6. Stability, Repeatability, Reproducibility 

Long term storage and operational stability of the electrodes are important for reliable continuous measurements over long periods. The stability was studied by repeated monitoring the oxidation response of the MWCNT-SPE and Er-GR-SPE at +0.48 V in the presence of 50 µM BR. Between two consecutive measurements, the electrodes were stored at 4 °C for 24 h and again subjected to the same repetitive measurements. Both sets of experiments, on MWCNT and Er-GR modified electrodes, did not exhibit significant changes in the oxidation potential and the peak current that is inferred from the coefficient of variation (0.4 to 0.62 for MWCNT and 0.3 to 0.57 for Er-GR, respectively), which means that the modified electrodes are stable, not affected by the oxidation products, and can be used for repetitive series of measurements. However, for long term storage, the current response was reduced to 64% after four weeks for MWCNT-SPE and 72% for Er-GR-SPE, as shown in Figure 5C. Furthermore, to demonstrate the good reproducibility of the experimental results, six of each electrodes were fabricated and the amplitude of the oxidation current for 50 µM BR was compared. A SD of 3.6% and 2.85% for MWCNT and Er-Gr modified electrodes, respectively, was determined, which confirms that the measurements are highly reproducible.

### 3.7. Real Sample Analysis

In human serum, BR exists in two forms namely conjugated (free form) and unconjugated (BR-albumin complex) and, therefore, the influence of albumin concentration on free BR was studied. In the presence of 50 µM of albumin, the sensitivity of both, MWCNT-SPE (6 nA µM^−1^ cm^−2^) and Er-GR-SPE (13 nA µM^−1^ cm^−2^), decreased (Figure 5D shows the response for the Er-GR-SPE). A further increase of the albumin concentration up to 400 µM reduced the performance of the BR sensors less significantly. It suggests that most of the BR binds with albumin when adding 50 µM albumin, however, when increasing the concentrations, the amount of free BR remains the same [25]. Control measurements in the absence of BR did not show a significant response for albumin. The concentration of free BR was measured in a blood serum sample in the presence of 400 µM albumin, using a standard addition technique. A drop of the sample was placed on the nafion membrane coated Er-GR-SPEs and the corresponding current responses were measured. The accuracy of the measurement was studied by determining the recovery of known amounts of BR added to the samples. Therefore, the concentration of free BR was quantified for each addition of BR, as shown in Table 1, by interpolating the current response into the calibration curve. Each reading represents the average of three measurements. 

## 4. Discussion

The SEM characterization of the nanomaterials modified electrodes show that the functionalization of MWCNT on SPE and the electrochemical reduction of GO were successful. Cyclic voltammetric characterization in the presence of electrochemical probe (Fe^3+^/Fe^2+^) shows that graphene modified SPE exhibit higher redox currents than MWCNT modified SPE (Figure 3C). The higher electrochemical current response of the Er-GR-SPE can be explained by the enhanced electron transfer rate and a larger surface area. Using Laviron’s method [35], the electron transfer rates of the MWCNT-SPE and Er-GR-SPE were estimated, revealing that the Er-GR-SPE transfer 67 ± 9 electrons per second between the redox probe and carbon electrode, which is two times faster than for the MWCNT-SPE (34 ± 6 s^−1^). Furthermore, the surface area of the SPEs after nanomaterial deposition was estimated using Randles-Sevciks equation [36]:

(1) Ip=(2.69×105)An32D12Cγ12,

where D is the diffusion coefficient of K_3_[Fe(CN)_6_] in solution, which is 6.7 × 10^−6^ cm^2^ s^−1^, and C is the concentration of the redox probe (mol cm^−3^). The parameters *I_p_*, *n*, *γ* and *A* correspond to the maximum current response, the number of electrons participating in the redox reaction (for Fe^3+^/Fe^2+^, *n* = 1), the scan rate (V s^−1^) and the surface area (cm^2^). The calculated surface areas using Equation (1) follow the order of the bare SPE (0.069 cm^2^) < MWCNT-SPE (0.075 cm^2^) < Er-GR-SPE (0.09 cm^2^). These results clearly show that the Er-GR modified SPE exhibit a larger surface area than MWCNT. 

The electrocatalytic oxidation of BR and the following oxidation reactions are irreversible by nature as described in Equations (2)–(4) under our experimental conditions, which matches well with earlier reports [24,37].

(2) $$Bilirubin\overset{\sim +0.25\;V}{\to }Biliverdin$$

(3) $$Biliverdin\overset{\sim +0.48\;V}{\to }Purpurine$$

(4) $$Purpurine\overset{\sim +0.70\;V}{\to }choletelin$$

The electroanalytical performances (detection limit, linear range, and sensitivity) of the MWCNT-SPE and Er-GR-SPE are comparable with earlier reported methods [12,13,15,17,19,24,25,26,27,38,39,40], as shown in Table 2.

The influences of the interfering substrates on BR oxidation were negligible in both MWCNT-SPE and Er-GR-SPE, except AsA (Figure 5A,B). The strong effect of AsA was reduced using porous nafion membrane, enabling the selective BR measurement in real samples. It is evidenced from the literature that hydrogen peroxide does not interfere with BR measurements [27]. Furthermore, the interference with other peroxy-compounds was eliminated using an anionic nafion membrane, which efficiently suppresses the effect of negatively charged substrates. The good recovery values from 94% to 106.5% indicate a very good accuracy (Table 1). The dynamic linear range of the nanomaterial modified SPEs strongly favours the applicability of the present BR sensors for point-of-care BR measurements. 

## 5. Conclusions

A low-cost, reliable, and miniaturized point-of-care electrochemical sensor for BR has been successfully demonstrated using SPE functionalized with MWCNT and Er-GR. The electrochemical oxidation of BR on nanomaterial modified SPE was studied in detail. The observed results strongly support the non-enzymatic methodology and allow for measurements of BR in a wide range of concentrations, with a low limit of detection. Nanomaterial modified electrodes offer higher electron transfer rates than bare SPE, enabling measurements with high sensitivities in the nA µM^−1^ cm^−2^ range. The Er-GR-SPE exhibits better electroanalytical performances than MWCNT-SPE; this is attributed to the higher electrical conductivity. The selectivity of the BR sensor towards biological species was ensured by eliminating the suspecting interfering substrates using a negatively charged nafion membrane. Finally, the analytical applicability of the assay was validated with human blood serum samples. We conclude that the present SPE based BR sensors can be highly beneficial for high quality healthcare management and could be suitable for point-of care diagnosis.

## Figures and Tables

**Table 1:** Measurement of BR in human blood serum samples.

Human Blood Serum	BR Added (µM)	BR Found (µM)	Recovery (%)	RSD (%)
Sample 1	5	5.08	101.6	2.4
Sample 2	10	9.72	97.2	1.9
Sample 3	15	14.10	94.0	3.1
Sample 4	20	21.3	106.5	2.0
Sample 5	25	24.3	97.2	1.4
Sample 6	30	28.7	95.6	2.7

**Table 2:** Comparison of the electroanalytical parameters of the present electrochemical sensors with earlier reported sensors.

Sensing Element	Linearity Range (µM)	Detection Limit	Sensitivity (µA µM^−1^ cm^−2^)	Reference
BOx/SiO_2_@ZrONPs/Au	0.02–250	0.1 nM	---	Batra_2013
PolyTTCA–Mn(II)	0.1–50	40 ± 3.8 nM	---	Rahman_2008
MWCNT/SPE		4.2 ± 0.1 µM	52.2 ± 2.4	Taurino_2013
BOx/Pt	100–200	8 µM	---	Klemm_2000
FcAI/NG/MWCNT/GCE	1–100	0.12 µM	---	Wang_2009
PSS-RGO/GCE	0–450	2	0.16	Balamurugan_2015
BOx/GONP@Ppy/FTO	0.01–500	2.6 nM	---	Chauhan_2016
BOx/MWCNT/GNs/AuNPs/GCE	1.33–71.56	0.34 µM	0.327	Feng_2013
BOx/AuNPs/MPTS/Au	1–5000	1.4 nM	---	Kannan_2011
Nafion/Mn-Cu/GCE	1.2–420	25 ± 1.8 nM	---	Noh _2014
HSA/AuNCs/ITO	0.2–7	86.32 nM	0.34	Santhosh_2016
HAP/QC	0.05–80	0.01 µM		Yang_2011
Er-GR/SPE	0.1–600	0.1 ± 0.018 µM	0.030	Present work
MWCNT/SPE	0.5–500	0.3 ± 0.022 µM	0.015	Present work

Box—Bilirubin oxidase; ZrONPs—Zirconium oxide nanoparticles; PolyTTCA—Poly-terthiophene- 3-carboxylic acid; FcAI—Ferrocene carboxamide; NG—Gold nanoparticles; GCE—Glassy carbon electrode; PSS-RGO—Poly styrene sulfonic acid-reduced graphene oxide; GONP@Ppy—Graphene oxide nanoparticle@polypyrrole; FTO—Fluorine doped tin oxide; GNs—graphene; MPTS—(3-mercaptopropyl)-trimethoxysilane; HAS—Human serum albumin; AuNC—Gold nanoclusters; HAP—Hydroxyapatite; QC—Quartz crystal.
